# A Study on Renal Failure Management in Patients Diagnosed With Multiple Myeloma

**DOI:** 10.7759/cureus.47460

**Published:** 2023-10-22

**Authors:** Lama M Almuhaysen, Amal Badr Abu Al Alaa

**Affiliations:** 1 Internal Medicine, Prince Sultan Military Medical City, Riyadh, SAU

**Keywords:** neoplastic disorder, survival rate, management, multiple myeloma, renal failure

## Abstract

Background: Renal impairment, often occurring as a result of multiple myeloma, poses a significant risk to patient health and quality of life. Therefore, the study was conducted with the objective of investigating the management of renal failure in patients diagnosed with multiple myeloma in Saudi Arabia.

Methods: A retrospective observational study was conducted during the period from January to August 2023 for the duration of eight months. The data were collected from the patients diagnosed with multiple myeloma, available from the medical records of Prince Sultan Military Medical City, Saudi Arabia.

Results: The data were collected from a cohort of 85 patients with multiple myeloma. Among patients, males were 62 (72.94%), and females were 23 (27.06%). The mean (SD) age was 68.43 (7.24) years. Among the cohort, 42 (49.1%) were International Staging System (ISS) stage III, and 32 (37.6%) were light chain myeloma. Most patients underwent novel agent-based treatment regimens (a combination of immunomodulator, proteasome inhibitor, and monoclonal antibody; 59, 69.41%), followed by conventional therapy; 16, 18.82%). Multivariate analysis of patient survival showed significant associations with three variables: increased calcium level (P=0.007), eGFR <30 mL/min (P=0.004), and novel agent induction (P=0.001).

Conclusion: This study sheds light on the intricate management of renal failure in multiple myeloma patients, specifically within the Saudi population. The observed associations between survival and various variables emphasize the critical role of renal function in overall patient outcomes.

## Introduction

Renal failure, a complex and challenging condition, has garnered significant attention within the medical community due to its intricate relationship with various underlying diseases [1]. Renal impairment, often arising because of multiple myeloma, poses a considerable threat to patients' health and quality of life [2]. The intricate interplay between these two conditions has prompted a growing need for comprehensive research, particularly within specific population groups, such as the Saudi population [3].

Multiple myeloma, a neoplastic disorder of plasma cells, has shown an increasing incidence globally, with regional variations necessitating tailored management strategies [4]. In Saudi Arabia, the prevalence of multiple myeloma has been reported to be on the rise, warranting a focused investigation into its associated complications, particularly renal failure. In 2016, the incidence of multiple myeloma in Saudi Arabia was reported to be 265 with 206 deaths [5]. The interrelation between multiple myeloma and renal function is multifaceted [6]. However, despite the significance of this association, only limited studies have studied the nuances of renal failure management in multiple myeloma, especially in the Saudi population.

The main etiological cause of renal impairment in multiple myeloma patients is due to the enhanced release of overproduction of nephrotoxic Ig and, to a lesser extent, non-Ig causes are also involved. In the case of Ig-related etiology, cast nephropathy is the major cause and contributes to 40-63%, and light chain deposition disease (LCDD) contributes to 20-25% and amyloidosis (15-35%) [7,8]. The intricate relationship between these conditions is further complicated by the potential for disease progression, necessitating a comprehensive understanding of the underlying mechanisms to develop effective management strategies [9].

In recent years, advancements in therapeutic approaches have revolutionized the landscape of multiple myeloma management [10]. Targeted therapies, immunomodulatory agents, and proteasome inhibitors have shown promise in enhancing patient outcomes and extending survival rates. However, the effect of these treatments in managing renal function remains a subject of ongoing investigation [11]. The delicate balance between achieving optimal disease control and preserving renal health poses a clinical dilemma, further underscoring the need for evidence-based guidelines specific to the Saudi population.

While a plethora of research exists [12], few studies have centered their focus on the unique attributes of the Saudi population. Factors such as genetic predisposition, environmental influences, and healthcare infrastructure may contribute to varying disease patterns and treatment responses [13,14]. Therefore, a comprehensive exploration of managing renal failure in Saudi patients of multiple myeloma is warranted, aiming to bridge the knowledge gap and provide valuable insights for clinicians and researchers alike [15,16].

This research article aims to address the gap by delving into the intricate relationship between renal failure and multiple myeloma in the Saudi population. Through this research, we aspire to contribute valuable insights that can inform clinical decision-making, improve patient care, and ultimately enhance the overall prognosis for Saudi individuals battling the complex synergy of both renal failure and multiple myeloma. Therefore, the study was conducted with the objective of studying renal failure management in patients diagnosed with multiple myeloma in Saudi Arabia.

## Materials and methods

Study design

A retrospective observational design was employed to examine the management of kidney failure that occurs in multiple myeloma patients. The data were collected from the patients diagnosed with multiple myeloma, available from the medical records of Prince Sultan Military Medical City, Saudi Arabia, during the period from January to August 2023.

Sample selection

The study aimed to investigate renal failure management specifically in patients diagnosed with multiple myeloma. Therefore, the inclusion criteria encompass patients with both multiple myeloma and renal impairment aged 18 years and above having all available medical records to ensure the relevance of the findings to the research objectives. Exclusion criteria were established to maintain the focus on the target population and to ensure that the results accurately reflect the impact of multiple myeloma on renal failure management in the specified context. Patients with renal impairment due to causes other than multiple myeloma, patients with incomplete or insufficient medical records, and patients below 18 years of age were excluded from the study.

Data collection

Demographic and clinical data: Relevant demographic information (e.g., age, gender) and clinical characteristics (e.g., disease stage, treatment history, laboratory results) of the included patients were collected from medical records.

Management approaches: Detailed information on the management approaches employed for kidney failure in multiple myeloma patients was also collected. This includes documentation of supportive care measures, chemotherapy regimens, immunomodulatory agents, targeted therapies, and the utilization of renal replacement therapy.

Treatment outcomes: Data on treatment outcomes, such as renal function improvement (ml/min), response to therapy, and overall survival rates, were extracted from the medical records.

Data analysis

The data were analyzed with Statistical Product and Service Solutions (SPSS) (version 25; IBM SPSS Statistics for Windows, Armonk, NY). Descriptive statistics were used to summarize the demographic and clinical characteristics of the patient sample. The management approaches utilized for kidney failure were reported as frequencies and percentages. Treatment outcomes were analyzed using survival analysis techniques, such as Kaplan-Meier curves and Cox regression models, to assess factors influencing patient outcomes.

Ethical considerations

This study adhered to the ethical guidelines and regulations for using patient data. IRB approval from Prince Sultan Military Medical City, Saudi Arabia (number 1825), was obtained from the relevant authorities before data collection. Patient confidentiality and data anonymity were ensured throughout the research process.

## Results

The data collection was done from 85 patients with multiple myeloma. Among 85, males were 62 (72.94%), and females were 23 (27.06%). The mean (SD) age was 68.43 (7.24) years. Most of them were International Staging System (ISS) stage III (42, 49.41%) and light chain type (32, 37.6%). All these data and the mean (SD) values of various biochemical parameters, such as serum creatinine, hemoglobin, albumin, macroglobulin, etc. were illustrated in Table 1.

**Table 1:** Demographics and clinical characteristics of the study participants (n=85).

Parameters	Number (%)
Age in years (mean±SD)	68.43±7.24
International staging system (ISS) stage, n (%)	
I	15 (17.65%)
II	28 (32.94%)
III	42 (49.41%)
Gender (n, %)	
Male	62 (72.94%)
Female	23 (27.06%)
Multiple myeloma type (N (%))	
Light chain	32 (37.6%)
Immunoglobulin G (IgG)	25 (28.41%)
Immunoglobulin A (IgA)	18 (21.18%)
Immunoglobulin M (IgM)	2 (2.35%)
Biclonal	5 (5.88%)
Non-secretory	3 (3.53%)
Serum Creatinine(mg/dl) (mean±SD)	3.12±0.95
Serum Hemoglobin (g/dl) (mean±SD)	9.12±1.72
Serum calcium (mg/dl) (mean±SD)	10.54±2.65
β2 Microglobulin(mg/dl) (mean±SD)	9.32±1.44
Urine albumin (g/dl) (mean±SD)	1.65±0.06
Serum uric acid (μmol/L) (mean±SD)	534.76±42.12
Lactate dehydrogenase (U/L) (mean±SD)	215.32±34.12
eGFR (Glomerular filtration rate) mL/min, (mean±SD)	17.83±2.53
Creatinine clearance (ml/min) (mean±SD)	12.42±0.98
Urea (mg/dl) (mean±SD)	137.92±65.28
Bence-Jones proteinuria (n, %)	28 (32.94%)
Dialysis requirement (N (%))	32 (37.65%)
Mortality (N (%))	38 (44.70%)

Most patients underwent novel agent-based treatment regimens (a combination of immunomodulator, a proteasome inhibitor, and monoclonal antibody; 59, 69.41%), followed by conventional therapy (drugs such as cyclophosphamide, doxorubicin, melphalan, etoposide, cisplatin, and bendamustine; 16, 18.82%). Supportive care and novel agent-based regimens followed by ASCT were observed in seven (8.23%) and three (3.53%), respectively (Table 2).

**Table 2:** Treatment regimens in the present study.

Treatment Regimens	MM patients with renal failure (n=85)
Supportive care	7 (8.23%)
Conventional chemotherapy	16 (18.82%)
Novel agent-based regimens	59 (69.41%)
Novel agent-based regimens followed by autologous stem cell transplant (ASCT)	3 (3.53%)

A complete response to treatment was found among 47 (55.29%) patients. Partial and no renal responses were observed in 12 (14.12%) and 26 (30.59%) patients, respectively. These data are given in Table 3.

**Table 3:** Renal responses among the multiple myeloma renal failure patients based on IMWG criteria.

Renal responses	Multiple myeloma patients with renal failure (n=85)
Complete	47 (55.29%)
Partial	12 (14.12%)
No renal responses	26 (30.59%)

Univariate analysis of the study participants showed statistically significant associations with increased LDH, ISS III, increased calcium levels, eGFR<30 mL/min, novel agent induction, and age more than 70 years. However, multivariate analysis after adjusting for the confounding factors showed significant associations with three variables, namely, increased calcium level (P=0.007), eGFR <30 mL/min (P=0.004), and novel agent induction (P=0.001), is statistically significant in both univariate and multivariate analyses (Table 4).

**Table 4:** Univariate and multivariate analyses for the factors associated with overall survival.

Variables	Univariate analysis			Multivariate analysis		
	HR	95% CI	P value	HR	95% CI	P value
Increased Lactate dehydrogenase	2.12	1.75-3.87	0.004	1.87	1.54-2.76	0.08
International Staging System III	1.87	1.45-3.12	0.001	0.98	0.56-1.43	0.17
Increased calcium level	2.42	1.65-3.89	<0.001	2.34	1.42-3.52	0.007
eGFR (Glomerular filtration rate) <30ml/min	1.98	1.53-3.05	<0.01	1.82	1.32-2.86	0.004
Novel agent Induction	1.65	1.12-2.43	<0.001	1.34	0.87-1.76	<0.001
Age >70 years	2.76	1.83-3.24	<0.01	2.42	1.68-2.87	0.03

Among 85 study participants who were included in the study, mortality was observed in 38 patients. Among those who died, seven were taking supportive treatment, 13 were in conventional treatment, and 18 took novel treatment. None of the patients who took novel treatment along with ASCT died.

**Figure 1:**
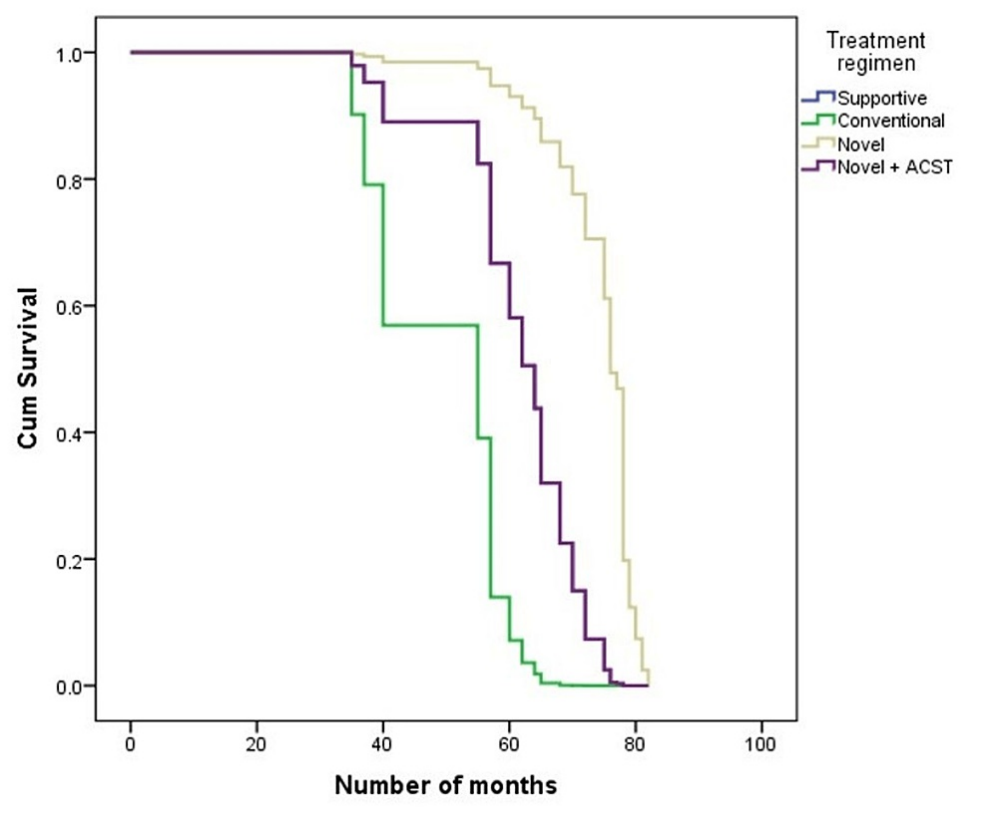
Survival curve for treatment regimens.

Similarly, the survival rate was found to be 0% in those who took supportive treatment, 18.8% in conventional treatment, 69.5% in novel treatment, and 100% in those taking novel treatment along with ASCT. The median survival in those who were taking supportive treatment was 28 months, conventional treatment was 34.50 months, novel treatment was 75 months, and novel treatment along with ASCT was 65 months. Differences were statistically significant (P=0.001) and illustrated in Figure 1.

**Figure 2:**
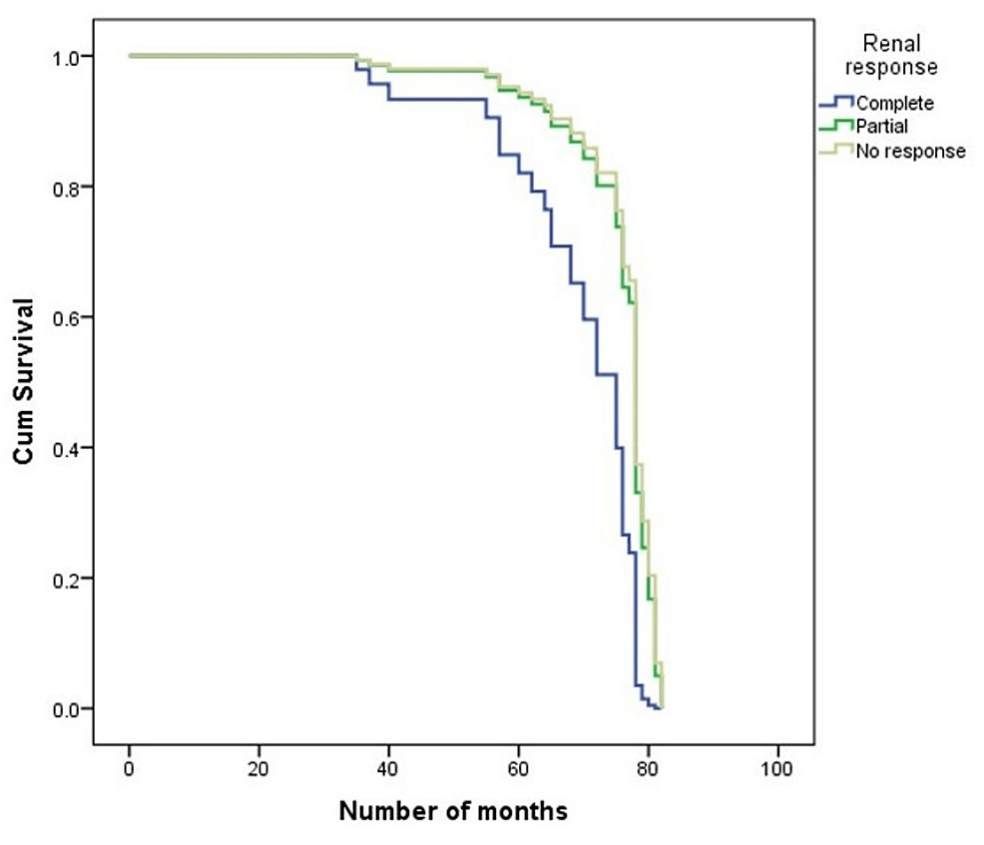
Survival curve for renal responses.

Approximately 61.7% of patients with complete renal response lived, 50% of patients with moderate renal response survived, and only 46.2% of patients with no renal response survived. The median survival in renal response (complete) was 62 months, renal response (partial) was 59 months, and no renal response was 42 months. Differences were statistically significant (P=0.041) and are illustrated in Figure 2.

## Discussion

The study aimed to investigate renal failure management in patients diagnosed with multiple myeloma in Saudi Arabia. Our findings provide valuable insights into the demographics, treatment modalities, and clinical outcomes, along with prognostic factors. The demographic profile of the study participants reflects a slightly higher representation of males, comprising 72.94% of the cohort. This distribution aligns with previous research, indicating a higher risk in males for multiple myeloma compared to females [17]. The mean age of 68.43 years observed in our study is consistent with the typical age range of multiple myeloma diagnosis [18]. Most patients were in ISS stage III, indicating an advanced disease state at the time of diagnosis [19]. Notably, the light chain type was prevalent, highlighting the relevance of monoclonal light chains in the pathogenesis of renal impairment [20].

Novel agent-based treatment regimens were the predominant therapeutic approach in our study population, representing 69.41% of patients. This trend aligns with the paradigm shift in multiple myeloma management, wherein immunomodulatory drugs and proteasome inhibitors, along with monoclonal antibodies, have demonstrated significant efficacy and improved patient outcomes [21]. Conventional therapy, although employed less frequently, remains an important consideration for selecting patients. Supportive care and ASCT were observed in a minority of cases, underscoring their role in specific clinical scenarios.

The treatment responses observed in our study underscore the complexity of managing renal failure in the context of multiple myeloma. Complete response was achieved in 55.29% of patients, indicating the potential for successful renal recovery with appropriate interventions. Partial renal responses were documented in 14.12% of cases, further highlighting the heterogeneity of treatment outcomes. Importantly, a significant proportion (30.59%) did not achieve renal response, underscoring the challenges associated with reversing renal impairment in some individuals. These findings emphasize the need for personalized treatment strategies tailored to each patient's clinical presentation and underlying disease characteristics.

The mean (SD) values of biochemical parameters provided valuable insights into the disease severity and its impact on renal function. Elevated serum creatinine levels were consistent with renal impairment, reinforcing the interconnectedness between multiple myeloma and renal dysfunction [22]. Hemoglobin levels and albumin levels, indicative of overall health and nutritional status, were also reflective of disease burden [23].

Multivariate logistic regression analysis yielded significant associations between overall survival and several variables. Increased calcium levels were identified as a significant prognostic factor, corroborating the role of hypercalcemia in disease progression [24]. Notably, an estimated eGFR <30 mL/min emerged as a powerful predictor of survival, emphasizing the critical impact on renal function patient outcomes [25]. The associations between novel agent induction and improved survival further underscore the therapeutic potential of these agents in managing both myeloma and renal complications [26].

Kaplan-Meier survival analysis revealed distinct survival rates based on renal response and treatment modality. Patients experiencing complete renal failure and undergoing conventional chemotherapy exhibited significantly lower survival rates. This observation supports the notion that renal response is closely linked to overall prognosis, highlighting the importance of effective renal management in enhancing survival outcomes [27,28].

The findings of this study have substantial clinical implications, providing a comprehensive understanding of renal impairment management in patients with multiple myeloma. The prevalence of renal impairment underscores the need for vigilant renal assessment and tailored interventions in order to get favorable patient outcomes [29,30]. The significant associations identified through multivariate analysis offer valuable insights into prognostic factors that can guide clinical decision-making. These findings warrant further exploration in larger cohorts and prospective studies to validate their robustness and potential application in clinical practice.

Limitations

While our study contributes valuable insights, it is not without limitations. The retrospective study design along with the small sample size may introduce selection bias and limit the generalizability of findings. Additionally, reliance on medical records for data collection, and possible variations in documentation practices across different healthcare centers may not fully capture the diversity of multiple myeloma presentations and treatment patterns within the Saudi population.

## Conclusions

This study sheds light on the intricacies of managing renal impairment in patients with multiple myeloma, specifically within the Saudi population. The interplay between disease characteristics, treatment modalities, biochemical parameters, and prognostic factors underscores the complexity of addressing renal impairment in the context of multiple myeloma. The observed associations between survival and various variables emphasize the critical role of renal function in overall patient outcomes. These findings underscore the importance of personalized treatment approaches that consider both myeloma-related and renal-specific factors. Further research is warranted to validate and expand upon these insights, ultimately advancing the management of renal failure in the complex landscape of renal impairment in multiple myeloma.
